# Tumor-educated platelet blood tests for Non-Small Cell Lung Cancer detection and management

**DOI:** 10.1038/s41598-023-35818-w

**Published:** 2023-06-08

**Authors:** Mafalda Antunes-Ferreira, Silvia D’Ambrosi, Mohammad Arkani, Edward Post, Sjors G. J. G. In ‘t Veld, Jip Ramaker, Kenn Zwaan, Ece Demirel Kucukguzel, Laurine E. Wedekind, Arjan W. Griffioen, Mirjam Oude Egbrink, Marijke J. E. Kuijpers, Daan van den Broek, David P. Noske, Koen J. Hartemink, Siamack Sabrkhany, Idris Bahce, Nik Sol, Harm-Jan Bogaard, Danijela Koppers-Lalic, Myron G. Best, Thomas Wurdinger

**Affiliations:** 1 Department of Neurosurgery, Cancer Center Amsterdam, Amsterdam UMC, VU University Medical Center, De Boelelaan 1117, 1081 HV Amsterdam, The Netherlands; 2 Cancer Center Amsterdam, Amsterdam, The Netherlands; 3 Brain Tumor Center Amsterdam, Amsterdam, The Netherlands; 4 Department of Pulmonary Medicine, Amsterdam UMC Location Vrije Universiteit Amsterdam, De Boelelaan 1117, Amsterdam, The Netherlands; 5 Department of Biomedical Data Science, Leiden University Medical Center, Leiden, The Netherlands; 6 Department of Medical Oncology, Amsterdam UMC Location Vrije Universiteit Amsterdam, De Boelelaan 1117, Amsterdam, The Netherlands; 7 Department of Physiology, Cardiovascular Research Institute Maastricht, Maastricht University, Maastricht, The Netherlands; 8 Department of Biochemistry, Cardiovascular Research Institute Maastricht, Maastricht University, Maastricht, The Netherlands; 9 Department of Laboratory Medicine, The Netherlands Cancer Institute – Antoni van Leeuwenhoek Hospital, Amsterdam, The Netherlands; 10 Department of Thoracic Surgery, The Netherlands Cancer Institute-Antoni Van Leeuwenhoek Hospital, Amsterdam, The Netherlands; 11 Department of Neurology, Amsterdam UMC Location Vrije Universiteit Amsterdam, De Boelelaan 1117, Amsterdam, The Netherlands; 12 Mathematical Institute, Leiden University, 2333 Leiden, CA The Netherlands

**Keywords:** Non-small-cell lung cancer, RNA sequencing, Diagnostic markers, Cancer

## Abstract

Liquid biopsy approaches offer a promising technology for early and minimally invasive cancer detection. Tumor-educated platelets (TEPs) have emerged as a promising liquid biopsy biosource for the detection of various cancer types. In this study, we processed and analyzed the TEPs collected from 466 Non-small Cell Lung Carcinoma (NSCLC) patients and 410 asymptomatic individuals (controls) using the previously established thromboSeq protocol. We developed a novel particle-swarm optimization machine learning algorithm which enabled the selection of an 881 RNA biomarker panel (AUC 0.88). Herein we propose and validate in an independent cohort of samples (n = 558) two approaches for blood samples testing: one with high sensitivity (95% NSCLC detected) and another with high specificity (94% controls detected). Our data explain how TEP-derived spliced RNAs may serve as a biomarker for minimally-invasive clinical blood tests, complement existing imaging tests, and assist the detection and management of lung cancer patients.

## Introduction

With more than two million new cases per year, lung cancer is one of the most commonly diagnosed type of cancer worldwide in both sexes, and the leading cause of tumor mortality^1^. A substantial proportion of this high lethality can be attributed to the often-late diagnosis of the disease, with metastasis being present at the time of diagnosis, leading to a 5-year survival rate of about 15%^2–4^. Earlier detection can drastically improve the chances of survival. For instance, a stage IA non-small cell lung cancer (NSCLC) patient who undergoes surgical resection has an estimated disease-free survival of 82% at 5-years^5,6^. Early detection is crucial to improve the outcome of the treatments for patients diagnosed with NSCLC (representing 85% of lung cancer cases^7^).

Screening tests may enable earlier identification of patients with NSCLC. Periodical chest X-rays and/or sputum cytology have been tested to screen subjects at high risk of lung cancer, but they failed due to low efficacy^8,9^. Alternatively, randomized clinical trials showed that low-dose computer tomography (LDCT) screening reduces the mortality of lung cancer in the high-risk groups^10–14^. The National Lung Screening Trial (NLST) reported a 20% decrease in lung cancer mortality using LDCT, in comparison to single-view chest radiography^13^. Furthermore, the Dutch-Belgian randomized lung cancer screening trial (NELSON) showed that spiral computer tomography (CT) screening reduces mortality of lung cancer by 26% in comparison to the not-screened Controls group in 10 years^10,11^. However, implementation of LDCT into clinical practice has been impaired so far by possible psychosocial distress of the patients, cost-effectiveness, and feasibility of the implementation of the screening process^9,15^.

Although tumor tissue assessment is the gold standard to confirm a lung cancer diagnosis, it represents only a temporal snapshot of the tumor mass inadequately reflecting its intra-tumor heterogeneity^16–19^. Liquid biopsy assays could provide minimally-invasive, real-time, and repeatable tests for screening, diagnosis, monitoring, molecular profiling, and prognosis of various tumor types, including NSCLC^16,18–20^. This type of test encompasses the molecular information from circulating tumor biomarkers isolated from body fluids such as blood and urine^19,21^. These circulating biomarkers include proteins^22^, circulating tumor cells (CTCs)^23–25^, cell-free DNA (cfDNA)^26–28^, circulating tumor DNA (ctDNA)^26,29^, cell-free RNA (cfRNA)^30^, extracellular vesicles^31,32^, and tumor-educated platelets (TEPs)^33–35^.

In the past years, TEPs have emerged as a promising source of biomarkers for liquid biopsy^33,34,36–43^. Several research studies have indicated that the transcriptomic and proteomic profile of platelets undergo alterations in response to the presence of NSCLC, suggesting their potential utility as a biomarker for the diagnosis and prognosis of NSCLC^44–54^. We have previously shown that the combination of a tailored RNA-sequencing and bioinformatics approach, termed thromboSeq^55^, enables the identification of spliced RNA signature in TEPs in cancer patients^37^. The thromboSeq pipeline was also successfully tested in several studies from our group and others^49,56–60^. Recently, we developed a TEP RNA-based blood test that enables the detection of 18 different cancer types with 99% of specificity, showing the potential of platelets to be used for blood-based cancer screening test^61^. Previously, a specific test for NSCLC patients detection was also generated using predominantly late-stage disease samples, leading to an accuracy of 81–88%^56^. Given the complexity and systemic nature of the advanced NSCLC stage, it remains unclear whether earlier stages of NSCLC can also be identified in the TEP RNA profiles.

In this study, we investigated the TEP RNA signatures of early and late-stage NSCLC patients for the development of a new diagnostic algorithm. We propose two different tests termed *HighSens* and *HighSpec* that can be applied to the detection and clinical management of NSCLC patients.

## Results

### Altered spliced RNA repertoire in TEPs

In this study, we included and analyzed 876 platelet samples collected from 466 NSCLC patients and 410 asymptomatic individuals (‘Controls’). Most of the NSCLC patients enrolled were diagnosed with adenocarcinoma or squamous cell carcinoma, ranging from stage I to stage IV (Supplementary Table S1). Blood was collected in 10 different hospitals and platelets were isolated according to the previously established thromboSeq protocol (Supplementary Figure S1a)^55^, which ensures minimal platelets activation and leukocyte contamination. No significant differences have been identified between platelet collected in different hospitals^55^. Total RNA from platelets was isolated, and RNA quality and quantity were evaluated before library preparation and RNA sequencing (Supplementary Figure S1b). After processing the raw sequencing data, bioinformatics analyses were performed employing our previously developed machine learning-based thromboSeq algorithm (see “Methods” section). Platelet RNA-sequencing libraries were analyzed using only intron-spanning (spliced) reads, to prevent any potential contribution from cell-free DNA contamination.

Next, samples were divided into training, evaluation, and validation series. Following predefined quality control steps (see “Methods” section), a total of 110 samples were excluded (Supplementary Figure S2a–d; Table 1), resulting in a total dataset of 399 NSCLC patients and 367 Controls (Table 1). We decided to allocate 20% of the samples to the training and evaluation series each, and the remaining 60% of the sample set to the validation series. The training series (n = 105) included 48 NSCLC and 57 control samples, the evaluation series (n = 103) included 47 NSCLC and 56 Controls samples, and the validation series (n = 558) included 304 NSCLC and 254 Controls.

**Table 1:** Cohort demographics and clinical information, including gender and age (median) of the NSCLC patients and asymptomatic individuals (controls). IQR, interquartile range. See also Table S1 for further details.

		Training series		Evaluation series		Validation series	
		NSCLC n = 48	Controls n = 57	NSCLC n = 47	Controls n = 56	NSCLC n = 304	Controls n = 254
Age	Years (IQR)	63(11)	64 (9.5)	63 (14)	65 (11)	63 (15)	42 (24)
Gender	Female (%)	21 (44)	26 (46)	24 (51)	30 (54)	141 (46)	159 (62)
	Male (%)	27 (56)	31 (54)	23 (49)	26 (46)	163 (54)	95 (38)

The training of the algorithm was performed using age-matched sample series to reduce the potential effect of age as a confounding factor to the classification algorithm. Although the deviation in the median age of the Controls not being ideal (median age: 64 (training), 65 (evaluation), 42 (validation)), the classification of each sample in the validation series is independent from the age of other samples included in this group. We compared the detection rates of the algorithm by selecting an age-matched sample subset from the validation series (median age: 53 (Controls), 65 (NSCLC Stage I-III), 63 (NSCLC Stage IV), data not shown), and confirmed similar rates as compared to the full dataset. Thus, although we cannot rule out at least some contribution of age of the individuals to the classification algorithm and its platelet RNA biomarker profile, it remains unlikely that age of the individuals contributed to the observed differences among the groups as a whole.

The NSCLC patients had on average 3928 different transcripts detected, whereas the Controls had 4031 transcripts detected (*p* < 0.001, Supplementary Figure S2e–f).

**Figure 1:**
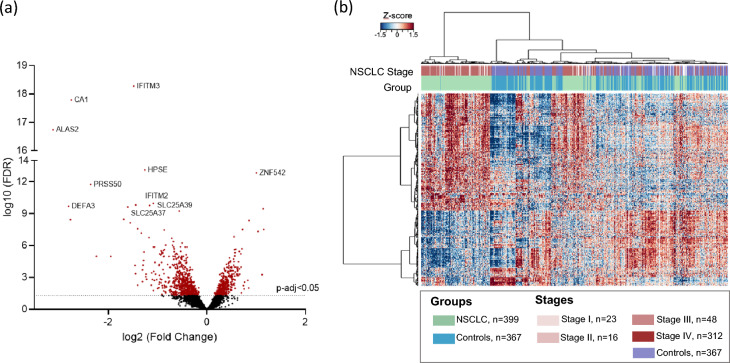
Overview of the samples included, and the platelet mRNA profiles from NSCLC and Controls. (**a**) Volcano plot illustrating (red) the 1090 mRNA differentially expressed spliced reads (FDR < 0.05). A total of 697 were significantly downregulated and 393 upregulated in the NSCLC group. (**b**) Heatmap with unsupervised clustering of the platelet mRNA profiles of NSCLC patient (green) and Controls (blue) groups included in all the series. Stages of the patients are indicated on the top of the heatmap according with the color code indicated in the legend on the right.

We searched for RNA sequences with differential splice junction reads by ANOVA analysis between all stages of NSCLC samples and Controls. In total 1090 RNAs were identified (FDR < 0.05), of which 697 were significantly downregulated and 393 upregulated in the NSCLC group (Fig. 1a). Unsupervised hierarchical clustering of these 1090 RNAs with differential splice junction reads resulted in a moderate separation between the two groups (Fig. 1b; *p* < 0.0001). We hereby confirmed our previous observation^56^ that patients with NSCLC have a differential platelet mRNA repertoire as opposed to Controls.

### PSO-enhanced thromboSeq algorithm for the detection of NSCLC patients

We previously developed an algorithm for the identification of predominantly stage IV NSCLC patients and non-cancer controls based on the differentially spliced platelet RNAs^56^, which was generated on NSCLC patients with advanced disease (only three stage I-II NSCLC patients were included). Here, we tested this NSCLC detection algorithm in a larger cohort of early-stage samples. Validation of 23 stage I, 16 stage II and 49 stage III samples resulted in poor detection rates in the earlier stages (detection rate stage I: 0%, n = 23; detection rate stage II: 6%, n = 16; detection rate stage III: 54%, n = 49; Supplementary Figure S3), indicating that the current NSCLC detection algorithm performs insufficiently for identification of individuals with earlier stages of the disease.

**Figure 2:**
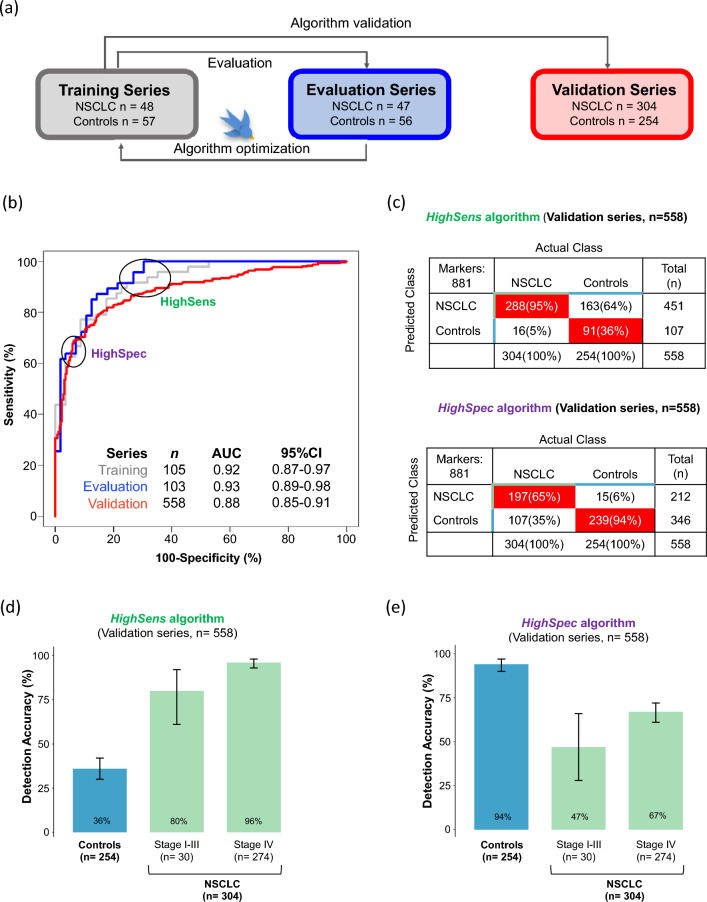
PSO-enhanced thromboSeq algorithm development, optimization, and validation, for the prediction of NSCLC and Controls using the HighSens and HighSpec algorithms. (**a**) Schematic representation of the samples series used to develop the PSO-enhanced thromboSeq algorithm. NSCLC and Control samples were divided into three different groups: Training (grey), Evaluation (blue) and Validation (red) series. Training and Evaluation series were employed for algorithm training and optimization. An independent cohort of samples (Validation series) was used to evaluate the performance of the test. (**b**) Receiver operating characteristic (ROC)-curves of the thromboSeq algorithm of the Training (grey line), Evaluation (blue line), and Validation (red line) series. Indicated are the sample number per series (n), Area Under the Curve (AUC) values and the 95%-confidence intervals (CI). The HighSens algorithm corresponds to a high sensitivity threshold setting and HighSpec to a high specificity threshold setting based on the Evaluation series. (**c**) Detection accuracy for the Control group and per NSCLC stage of the HighSens and the HighSpec algorithms, in the Validation series (**d**). Detection accuracy of the HighSens test in the Validation series. (**e**) Detection accuracy of the HighSpec test in the Validation series. Error bars indicate the 95% confidence interval calculated by the binom.test-function with Clopper-Pearson intervals implementation.

Due to these poor results, we decided to improve the detection of TEP-RNA signatures for early-stage disease by re-training the algorithm including more samples from patients diagnosed with early-stage NSCLC. Again, we employed training, evaluation, and an independent validation series to assess the performance and reproducibility of the test (Fig. 2a). In order to minimize potential confounding factors from demographic and clinical variables, the training and evaluation series were stage-, age- and gender-matched, (Supplementary Figure S4). The training series (n = 105) included 48 NSCLC and 57 control samples, the evaluation series (n = 103) included 47 NSCLC and 56 Controls samples, and the validation series (n = 558) included 304 NSCLC and 254 Controls (Table 1; Supplementary Table S1). The algorithm employs separate training and evaluation series to iteratively search for an optimal RNA biomarker panel selection separating both conditions (NSCLC and Controls) followed by a machine learning-based classification methodology^55^. Following optimization of the RNA biomarker panel, the newly trained algorithm is validated using the independent validation series.

After algorithm development, the classifier included an RNA biomarker panel of 881 markers, out of the 4082 spliced RNAs identified in the platelets, resulting in an area under the curve (AUC) of 0.92 in the training series (95% CI 0.87–0.97, n = 105), AUC of 0.93 in the evaluation series (95% CI 0.89–0.98, n = 103), and an AUC of 0.88 in the validation series (95% CI 0.85–0.91, n = 558; Fig. 2b; Table 2). As exploited in several other studies using tissue or blood-based classification algorithms, the algorithms use high dimensional RNA-sequencing data as input to directly classify individuals^62–64^. The large gene panel selection and bioinformatic analysis is an advantage to measure many potential biomarkers at once, and can overcome the limitation of targeted approaches such as qPCR and targeting sequencing.

**Table 2:** The performance of the PSO-enhanced thromboSeq algorithm per series. AUC, Area under the curve; CI, Confidence interval; n, number of samples, PR, predictive rate.

	HighSens algorithm	HighSpec algorithm
Training		
AUC (95% CI; n)	0.92 (0.87–0.97; 105)	
Specificity	67%	96%
Sensitivity	94%	44%
Evaluation		
AUC (95% CI; n)	0.93 (0.89–0.98; 103)	
Specificity	70%	98%
Sensitivity	100%	62%
Validation		
AUC (95% CI; n)	0.88 (0.85–0.91; 558)	
Specificity	36%	94%
Sensitivity	95%	65%
PR- Stage I (95% CI; n)	50% (0.19–0.81; 10)	10% (0.002–0.44; 10)
PR- Stage II (95% CI; n)	80% (0.28–0.99; 5)	60% (0.15, 0.95; 5)
PR- Stage III (95% CI; n)	100% (0.78–1.00; 15)	67% (0.38–0.88; 15)
PR- Stage IV (95% CI; n)	96% (0.93–0.98; 274)	67% (0.61–0.72; 274)

### Different clinical applications of the NSCLC detection algorithm

Subsequently, we here propose different clinical scenarios in which the NSCLC detection algorithm may be employed. The first application, termed *HighSens* test, aims to reduce the number of false-negative outcomes of the test. This type of test is designed to have a high level of sensitivity at the expense of specificity. This may be particularly useful for screening high-risk (e.g. heavy smokers) individuals to improve the detection of people developing the disease. The second application, termed *HighSpec* test, aims to avoid false-positive outcomes of the test. This type of test is designed to have high specificity at the expense of sensitivity. It can, for example, be used for screening purposes in the general population by adding a blood test as an adjunct to an imaging-based first-line screening tool. The inclusion of a blood platelets test may reduce the number of non-cancer individuals undergoing additional invasive diagnostic procedures. In order to reach both potential clinical applications, we employed the quantitative detection score (termed TEP-score ranging from 0 to 1^59^) that represents the confidence of the algorithm’s classification.

Based on the results obtained in the ROC-curve of the evaluation series, we defined an optimal cut-off TEP-score of 0.263 and 0.744 respectively for the *HighSens* and *HighSpec* test (Fig. 2b, c, Supplementary Figure S5, S6, and S7).

Application of these cut-off TEP-scores in the validation series resulted in a sensitivity of 95% and a specificity of 36% for the *HighSens* test (n = 558; Fig. 2c). By applying this cut-off, we detected 80% (95% CI 0.61–0.92 n = 30) of individuals diagnosed with early-stage disease (including stage I, II, III) and 96% (95% CI 0.93–0.98, n = 274) with advanced-stage of NSCLC (Fig. 2d, Supplementary Figure S6). Applying this test, we observed only 16 (5%) false negatives which were derived from five stage I, one stage II, and ten stage IV patients.

Application of the *HighSpec* cut-off TEP-score resulted in a specificity of 94%, corresponding to the correct identification of 239 out of 254 asymptomatic individuals (Controls), and a sensitivity of 65% in the validation series (Fig. 2c). Accuracy in the detection of stages I, II, and III was 47% (95% CI 0.28–0.66, n = 30) and 67% (95% CI 0.61–0.72, n = 274) for stage IV (Fig. 2e, Supplementary Figure S7).

The majority of the NSCLC patients with a stage III tumor included in this study (n = 48) are stage IIIa (Supplementary Table S1), which means that these tumors are locally-advanced and, at these earlier stages of disease therapy and prognosis is far different from stage IIIb. Therefore, we decided to further explore the detection rates for these sub-groups and observed that in the HighSens test, both patients with a IIIa and IIIb stage tumors had a detection rate of 100%. Using the HighSpec test, patients with stage IIIa had a detection rate of 60%, where-as those with stage IIIb of 67% (Supplementary Figure S8).

The detection rate of the Controls and NSCLC group was consistent, independent of the samples being from male or female individuals, which demonstrates that the classification is not biased by the gender of the individuals (Supplementary Figure S9). Random sampling of alternative training and evaluation series (n = 1000 iterations) resulted in similar classification accuracies (AUC validation series: 0.94, IQR: 0.02), whereas assigning random diagnostic group labels to the samples in the training series, expecting non-sense random classifications, results in diminished classification accuracies (n = 1000 iterations; AUC: 0.49, IQR: 0.19).

Analysis of our current dataset separated for smoking status, showed that there is a higher detection accuracy on NSCLC patients who are smokers, employing the *HighSpec* test (77% in smokers versus 62% in former smokers or never/unknown smokers). We hypothesize that smoking may be an additional confounding factor in this biomarker development process that requires attention in follow-up studies (Supplementary Figure S10).

Lastly, we compared our 881 RNA biomarker panel with previous publicly available studies using TEPs as an RNA biosource for cancer biomarkers. From this analysis, we observed an overlap of 270 RNAs (32,53%) with the previous NSCLC thromboSeq algorithm^56^ indicating that other platelet RNAs might be required to detect earlier-stage NSCLC samples (Supplementary Figure S11). Interestingly, we also found an overlap of 22 genes with the 48 gene panel from Sheng et al*.*, where TEPs RNA-sequencing data of NSCLC patients (*n* = 402) and Controls (*n* = 231) were analyzed in the Gene Expression Omnibus using an SVM classifier. This study performed differential gene expression analysis using minimal redundancy, maximal relevance (MRMR) method, and the optimal biomarker panel was selected using Incremental Feature Selection (IFS)^49^. Although our study design differed from Sheng et al*.,* we could confirm the significant deregulation of two genes, IFITM3 and HPSE, found in their study as potential biomarkers for NSCLC.

## Discussion

Application of liquid biopsies as diagnostic tool may advance earlier detection of cancer, thus, creating the possibility for prompt treatments and an increase in the survival of the patients. TEPs are promising sources of liquid biopsies, as shown by our group and others, however, the performance of the previous NSCLC TEP-based algorithm to detect early-stage of the disease has not been fully explored. In the previous thromboSeq NSCLC classifier^56^, out of 402 samples only three were collected from patients diagnosed with stage I (n = 1) and II (n = 2), making the algorithm more suitable for the detection of advanced-stage cancer patients. Here, we observed that the inclusion of earlier-stage NSCLC samples into the algorithm training process is key to improving detection rates in these locally-advanced (I-III) samples, without considerably reducing the detection rates of late-stage (IV) disease. With this newly trained algorithm, we obtained an AUC of 0.88 (95% CI 0.85–0.91; n = 558) in an independent validation series (Fig. 2b). We propose two possible scenarios on how this algorithm could be implemented as a pre-clinical test for blood-based NSCLC diagnostic. Adjusting the threshold settings of the TEP-score, we have defined two different tests termed *HighSpec* and *HighSens*. Though the current study cohort is suboptimal for modeling these differential study populations, it exemplifies the possible directions that can be taken with this blood test.

**Figure 3:**
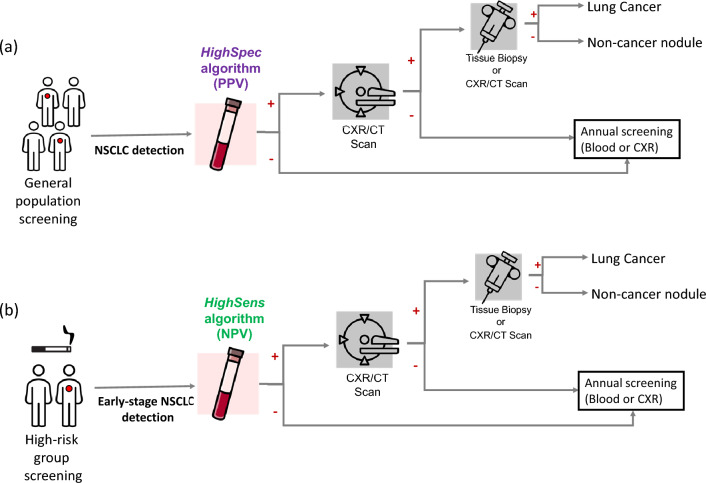
Schematic representation of the clinical practice for lung cancer screening and follow-up and the proposed approach for the application of the TEP HighSens and HighSpec blood tests. CXR, chest X-ray; CT, computed tomography; PPV, optimal positive predicted value; NPV, optimal negative predicted value.

The *HighSpec* test has a high specificity and was optimized to have an optimal positive predictive value (PPV) and aims to reduce false positives when screening the general populations for the detection of NSCLC (Fig. 3a). In comparison with the imaging tests for NSCLC screening^65^, liquid biopsy-based tests may be more advantageous as they are easier to implement for large-scale testing due to less demanding logistics. Limiting the number of people requiring an imaging test will also likely result in reducing the costs and pressure on the healthcare system. Dedicated cost-effectiveness studies are required prior to implementing the blood tests in such clinical routine. With our algorithm, only 6% of the asymptomatic Controls have been classified as cancer patients (false positive). Furthermore, the test may detect 65% of cancer cases in the general population in a non-invasive screening test, enabling faster treatment and improved patient outcomes.

We here suggest that other future studies could test the *HighSpec* test in a cohort of patients with suspicious lung nodules to assess its utility in differentiating patients with pulmonary nodules from those with an early-stage NSCLC. Distinguishing benign pulmonary nodules from lung cancer (especially at early-stages) is challenging in the clinic due to the low specificity of low-dose CT (high false-positive rates up to 96%)^8,66^ This test may help clinicians to determine the best follow-up time for the patients who had a previous positive CT-scan. In case of a negative blood test, the date of the follow-up appointment could be extended (for example, after 6 months instead of the usual 3 months)^67,68^. Reducing the need for such frequent CT-scans, will diminish the radiation exposure of the patient, reduce the medical costs, and the pressure on the healthcare system due to frequently scheduled follow-ups of individuals with benign lung nodules. The utility of such a blood test may also reduce the need for invasive tissue biopsies.

On the other hand, the *HighSens* cut-off allowed the development of a highly sensitive NSCLC test with an optimal negative predictive value (NPV) that aims to reduce false negatives and detect early-stage NSCLC (Fig. 3b). Combining this test with LDCT screening of high-risk populations (such as smokers) could improve the early detection of cancer patients with the advantage of large-scale testing. This blood test could complement the CT screening, giving a molecular insight into the imaging testing and the possibility of detecting an anomaly even when the tumor is in its early phase (e.g., small tumor size) (Fig. 3b).

In the future, clinical validation using a cohort of smokers is necessary, as well as assessing its combination with imaging tests for the design of best implementation settings. The inclusion of blood tests in studies similar to the NELSON trial^65^ and a health technology assessment (HTA) is necessary to ensure the cost-effectiveness of such design^69^. In this clinical scenario it would be interesting to also assess the direct comparison of the obtained blood TEP RNA signatures with the clinicopathological findings of the imaging test using chest X-ray (CXR) and/or computed tomography (CT) (Fig. 3), similar to the previous study done by our group with glioblastoma patients^59^.

With the *HighSpec* cut-off, the detection of stages I-III was relatively low (46%) when compared to more advanced stage. These observations can be partially explained by the ‘education’ process occurring in the platelets, as in the presence of a tumor, platelets transcriptome may undergo several changes^38,41–43,59^. It has been shown before that surgical resection of glioblastoma with concomitantly reduced tumor load results in a reduction of the TEP-score^59^. This observation can, at least partially, be due to a decrease in platelets ‘education’ together with the natural platelet turnover in 7–10 days leading to diminished alteration in TEPs RNA profiles. As a result, earlier stages of the disease (i.e., stages I and II) are more prone to be classified as false-negative likely due to their lower tumor loads and lower detection (TEP) scores. Lower tumor load could lead to fewer platelet-tumor interactions. It is likely that a blood sample taken from a subject with early-stage NSCLC, may contain a smaller percentage of platelets that have interacted with the tumor, leaving the majority of circulating platelets as ‘uneducated’. We have demonstrated previously in Sol et al. (2020)^59^, that the TEP-RNA profile of patients with a glioblastoma is associated with the tumor load and could be correlated with response measures. Whenever possible, samples from treatment naïve patients were included in our study cohort. The aim of this test is not to perform patient stratification but cancer detection. Even considering that the treatment may decrease cancer signal by reducing tumor load, our test was still accurate on detecting the patients.

For the false-positive samples, it is difficult to draw a precise assumption on why these samples are misclassified. It is known that smoking can lead to alteration in the transcriptome of platelets, by triggering inflammation^70^ and platelet activation^40,71–75^. Smoking-induced inflammation can also trigger lung cancer development^70^. Further studies need to be done to understand if smoking habits can lead to misclassification of the samples. Additionally, we did not perform any clinical examination to exclude the presence of cancer in the asymptomatic controls at the time of blood collection. Moreover, due to the anonymization of the control samples, clinical follow-up is not possible after the blood collection date. The asymptomatic individuals may therefore also have unnoticed cancer, which cannot be checked due to the anonymization of the samples. Further data and investigation would be needed to assess if these health conditions may have an impact on the algorithm to classify them as non-cancer samples.

A larger number of stage I-III NSCLC patients would be a relevant addition to this study, especially for validation of the algorithm. On a relevant note, the classification of samples in the validation series is independent from the number of samples included in this group and independent from algorithm development (training and evaluation series; see also Fig. 2d, e, Supplementary Figure S6e and S7e). This provides us strong evidence that the algorithm can identify earlier stages of lung cancer, since a balanced distribution of the number of samples with each tumor stage (I, II, III, IV) and also age and gender-matched sets between groups (NSCLC and Controls) were include in these series (Table 1, Supplementary Table S1). Indeed, validating additional earlier stage disease samples is of high-relevance for follow-up studies.

As noted previously, the selection of the cohort of samples can influence the performance of the algorithm. In our study, patients who have less commonly diagnosed histopathological types of lung cancer (e.g. carcinoid or sarcomatoid) were underrepresented. This situation likely caused the SVM-algorithm to be poorly trained for detecting those subtypes of lung cancer, though the algorithm was also not validated for these rare forms of lung cancer. The performance of the algorithm could also be further improved using non-cancer control samples collected from individuals with different health conditions, e.g. chronic inflammatory diseases, cardiovascular diseases^76^ and/or infectious diseases. To exclude potential bias associated with isolation location, the RUV correction was applied during the processing of the sequencing data. Additionally, in the future automated and standardized blood processing devices should also be implemented.

Several circulating biomarkers for early detection of NSCLC are being investigated such as cfDNA^27,28,77^, miRNA^78^, metabolites^79^, CTCs^80^, and exosomes^81^. The combination of information obtained from other circulating biomarkers and different platforms (e.g., NanoString^82^) should also be investigated. Our protocol has potential for such combinatorial studies, because other than the platelets, plasma is also stored and readily available for further analysis in the future.

## Conclusions

The thromboSeq PSO-algorithm enables the selection of an RNA biomarker panel (*n* = 881) and the validation of two blood tests, one with high sensitivity (95% NSCLC detected, n = 304) and another with high specificity (94% Controls detected, n = 254). The inclusion of a larger set of samples in the study cohort, could make the algorithm more robust and potentially decrease the number of false predicted samples. In the future the performance of the algorithm should also be tested in other types of cohorts, such as samples with smoking-habit, patients diagnosed with benign pulmonary nodules and chronic obstructive pulmonary disease (COPD). Functional assays based on liquid biopsy have already entered a molecular testing guideline for lung cancer. Currently when the access to a tissue biopsy is limited or insufficient for molecular testing, cfDNA analysis for detection of sensitizing *EGFR* mutations provides the information for a target treatment selection^14,83^. Additionally, platelets are triggered as first-responders to a tumor, whereas cfDNA is frequently just released at later stages of the disease. Overall, TEP-derived spliced RNA could potentiate minimally invasive blood tests, complementing the information obtained with imaging and tissue biopsies, and assisting clinicians in the management of lung cancer patients.

## Methods

### Clinical sample collection and platelet isolation

Peripheral blood samples were collected from NSCLC patients and individuals with no known cancer history (controls) at the Amsterdam University Medical Centers (VUMC and AMC locations, Amsterdam, The Netherlands), the Netherlands Cancer Institute – Antoni van Leeuwenhoek Hospital (Amsterdam, The Netherlands), the Utrecht Medical Center (Utrecht, The Netherlands), the Maastricht University Medical Center (Maastricht, The Netherlands), the Radboud University Medical Center (Nijmegen, The Netherlands), Umea University (Umea, Sweden), Medical University of Vienna (Vienna, Austria) and Massachusetts General Hospital (Boston, USA). The samples of patients and controls included in the present study were retrospectively collected. Whole blood samples from individuals ≥ 18 years were collected in EDTA-coated purple-capped BD Vacutainers (cat. n. 367863, BD). All individuals included in the study signed an informed consent for blood collection and blood platelet analysis. Samples were processed following two standard protocols for platelet isolation (due to availability of the samples and separate biobanking), using two-step centrifugation at room temperature^48,55^. At the Maastricht University Medical Center, the blood samples were centrifuged at 240 g for 15 min to obtain platelet-rich plasma (PRP). Iloprost (50 nM) was added to the PRP to minimize ex-vivo platelet activation. PRP was centrifuged for two minutes at 1600 g to spin down the platelets. RNAlater (Thermo Scientific) was added to the platelets pellet and stored at − 80 °C until further use. In all the other hospitals, the whole blood samples were centrifuged at 120 g for 20 min to separate the PRP from nucleated blood cells. PRP was then centrifuged at 360 g for 20 min to pellet the platelets. Platelet pellets were resuspended in RNAlater and, after overnight incubation at 4 °C, frozen at − 80 °C. Both protocols ensure the isolation of highly pure platelet pellets with minimum platelet activation and leukocyte contamination. No significant differences were observed between the two protocols.

### Clinical data and study cohort selection

NSCLC patients were diagnosed by clinical, radiological, and pathological examinations. The staging was determined according to the 8^th^ TNM edition of the Union for International Cancer Controls (UICC)/ American Joint Committee on Cancer (AJCC)^84^. The NSCLC group includes stage I, II, III and IV samples of patients with or without previous treatment history (i.e., chemotherapy, radiotherapy, immunotherapy, surgery). The records of the NSCLC patients were reported for demographic variables (i.e., patient age, gender, stage and type of tumor, smoking status, metastases, current and prior treatments, and co-morbidities). An extensive list of the characteristics of the NSCLC patients and asymptomatic individuals (Controls) included in the study can be found in Supplementary Table S1. For transgender individuals, the new gender was stated (*n* = 1, Male). Part of the samples were previously used in other studies and their raw data files are deposited in the NCBI GEO database (GSE89843 and GSE183635). The additional raw data files are deposited in the NCBI GEO under the GSE207586 accession number. The Controls were chosen from asymptomatic individuals with no known cancer history. However, no additional tests or follow-ups were performed to verify the cancer-free status of the individuals in the Controls group at the time of blood collection and afterward. The Controls and the NSCLC group were matched for stage, age and gender. This study was performed according to the principles of the Declaration of Helsinki and approved by the institutional review board and the ethics committee of each participating hospital. Clinical follow-up of non-cancer control individuals was not possible due to ethical and privacy policies.

### Blood platelet isolation, platelet RNA isolation, RNA amplification, and RNA-sequencing

Platelets were isolated within 48 h after blood draw by differential centrifugation, according to a previously published and standardized protocol^55,56^ (Supplementary Figure S1a), with minimal leucocyte contamination and platelet activation^55,56^. Platelets were subjected to RNA isolation, SMARTer mRNA amplification, TruSeq cDNA labeling, and RNA-sequencing of which all steps were quality-controlled by Bioanalyzer (Agilent Technologies) analysis, as described extensively in the recently published thromboSeq protocol^55^. In short, platelet RNA was extracted using the miRVana RNA isolation kit (Thermo Scientific, Waltham, MA, USA, cat. nr. AM1560). The quality of extracted total RNA was assessed using Bioanalyzer (Agilent Technologies) analysis with RNA 6000 Picochip (Agilent Technologies). High-quality platelet RNA was defined by RIN > 7 and/or distinct ribosomal peaks (Supplementary Figure S1b). A total of 500 picograms of platelet RNA was subjected to cDNA synthesis and amplification using the SMARTer Ultra Low V3 RNA Kit (Clontech, Takara Bio, Mountain View, CA, USA, cat. nr. 634,853, Supplementary Figure S1b). Quality assessment for cDNA was performed using the DNA High Sensitivity chip (Agilent Technologies). All the amplified cDNA was sheared by sonication (Covaris Inc.) and followed by labeling with index barcodes for Illumina sequencing using the Truseq Nano DNA Sample Prep Kit (Illumina). Labeled DNA quality was assessed using the DNA 7500 chip (Agilent Technologies) and Bioanalyzer (Agilent Technologies). High-quality samples (product sizes between 300 and 500 bp) were pooled and sequenced using the Illumina Hiseq 2500 platform (Illumina, San Diego, CA, USA).

### Processing of raw RNA-sequencing data

Sequencing reads in FASTQ-format were trimmed employing Trimmomatic (v. 0.22)^85^, quality-checked, and subsequently aligned to the reference human genome (hg19) employing STAR (v. 2.3.0)^86^. Reads were quantified employing HTSeq (v.0.6.1) guided by the Ensemble gene annotation version 75^87^, and only spliced RNA reads were selected for follow-up processing. All subsequent analyses were performed in R (v. 3.3.0) and R-studio (v. 0.99.902).

### Analysis of differential splice junctions reads

For analysis of differential splice junctions reads, the ANOVA-comparison was employed as described previously^55^. The ANOVA statistics results are summarized in a list of spliced RNAs with a corresponding logarithm fold-change (logFC), p-value and false discovery rate (FDR) values per transcript. Here, we employed particle swarm optimization (PSO) for optimal separation of samples in heatmap-clustering (Ward clustering, significance determined by p-value of Fisher’s exact test) by iteratively adjusting the FDR threshold (200 particles, 12 iterations).

### NSCLC detection algorithm development

Before the start of the analyses, the total dataset was subdivided into three different sample series: the training, evaluation, and validation series. The training and evaluation series were employed as reference groups for quality control analysis. A balanced distribution of the number of samples with each tumor stage (I, II, III, IV) and age and gender-matched sets between groups (NSCLC and Control) was include in these series (Table 1). The dataset was subjected to a low-read counts filtering step and quality-control steps, using the following elimination criteria: transcripts with insufficient read coverage (i.e. RNAs with < 30 intron-spanning reads in > 90% of the training and evaluation samples); detection of < 1500 genes (Supplementary Figure S2 b, d); and a correlation coefficient < 0.5 between samples (Supplementary Figure S2 a, c). These filtering steps excluded 110 samples (43 Controls and 67 NSCLC samples; 23 (21%) samples were excluded due to little RNAs detected (13 NSCLC and 10 Controls); 78 (71%) samples were excluded due to low (cross) correlation (47 NSCLC and 31 Controls); 9 (8%) samples were excluded due to low logCPM (logarithm- Count Per Million) (27 NSCLC and 2 Controls). A total of 766 samples were used for further processing. Remove unwanted variation (RUV)-correction was applied to exclude potential bias introduced by residual cell-free DNA and other variables, such as patient age and isolation location^55,56^, resulting in a normalized dataset.

For the development of the NSCLC classification algorithm, a PSO/ Support Vector Machine (SVM)-driven meta-algorithm for the selection of the most contributively RNAs was employed. The swarm-variables for the NSCLC algorithm were: ‘lib.size’, ‘fdr’, ‘correlatedTranscripts’, and ‘rankedTranscripts. The employed boundaries were − 0.1 to 1.0; 50–FDR < 0.005; 0.5 to 1.0; and 50–FDR < 0.005, respectively. The algorithm leverages the use of many candidate solutions (i.e. particles) and by adopting swarm intelligence, the algorithm continuously searches for the optimal solutions, ultimately reaching the most optimal fit^55,56^. The samples assigned to the training series were employed as reference for data normalization, biomarker panel identification, and SVM-algorithm training set. The samples assigned to the evaluation series were employed for SVM-algorithm read-out and swarm-based parameter optimization. Following algorithm training, the parameters were locked, and validation was performed in the independent validation set of donors blinded for diagnosis. The thresholds for the *HighSpec* and *HighSens* were selected from the classification score, which ranges from zero to one, and represents the classification score for either group. The *HighSens* threshold was selected from the range of evaluation series scores at which the classifier reached a sensitivity of 95% (Supplementary Figure S3) with the most optimal specificity, as determined by receiver operating characteristic (ROC)-analysis. Conversely, the *HighSpec* threshold was selected from the range of evaluation series scores at which the classifier reached a specificity of nearly 94% (Supplementary Figure S3) with the most optimal sensitivity, as determined by ROC-analysis. These thresholds were subsequently applied to the classification score of the independent validation series. Dependency of the SVM-algorithm classification based on the sample attribution, to either training or evaluation in the developmental series, was assessed by repeated (n = 1000) random allocation of samples into training or evaluation sets, while maintaining the RNA biomarker panel and the validation set. This should result in similar classification strength. To assess the random classification of the SVM-algorithm, class labels of the samples (‘NSCLC’ and ‘Controls’) were randomly permutated in the samples of the training set (n = 1000), while maintaining the RNA biomarker panel. This should result in a random classification (AUC ~ 0.5) and a lower predictive value^56^.

## Ethics approval and consent to participate

The research was conformed to the principles of the Helsinki Declaration and approved by the Ethics Committee of Amsterdam University Medical Centers (approval code: 11-4-117.4/pl, 2016.268 and 2017.545). All the participants have received and signed the informed consent for blood collection and blood platelets analysis.

## Supplementary Information

Supplementary Information 1.

Supplementary Information 2.

Supplementary Legends.

## Data Availability

The full software code is available via GitHub (https://github.com/MyronBest/thromboSeq_source_code_v1.5) and is for research purposes only. The raw sequencing data FASTQ-files generated and analyzed during the current study are available in the NCBI GEO database under accession numbers GSE183635 and GSE207586.
