# Supplementary material for: Tumor-educated platelet blood tests for Non-Small Cell Lung Cancer detection and management
Source: Sci Rep. 2023 Jun 8;13:9359. doi: 10.1038/s41598-023-35818-w (PMC10250384; doi:10.1038/s41598-023-35818-w)
Supplement: Supplementary file 1 — Supplementary Information 1. [file 41598_2023_35818_MOESM1_ESM.pptx]

## Slide 1
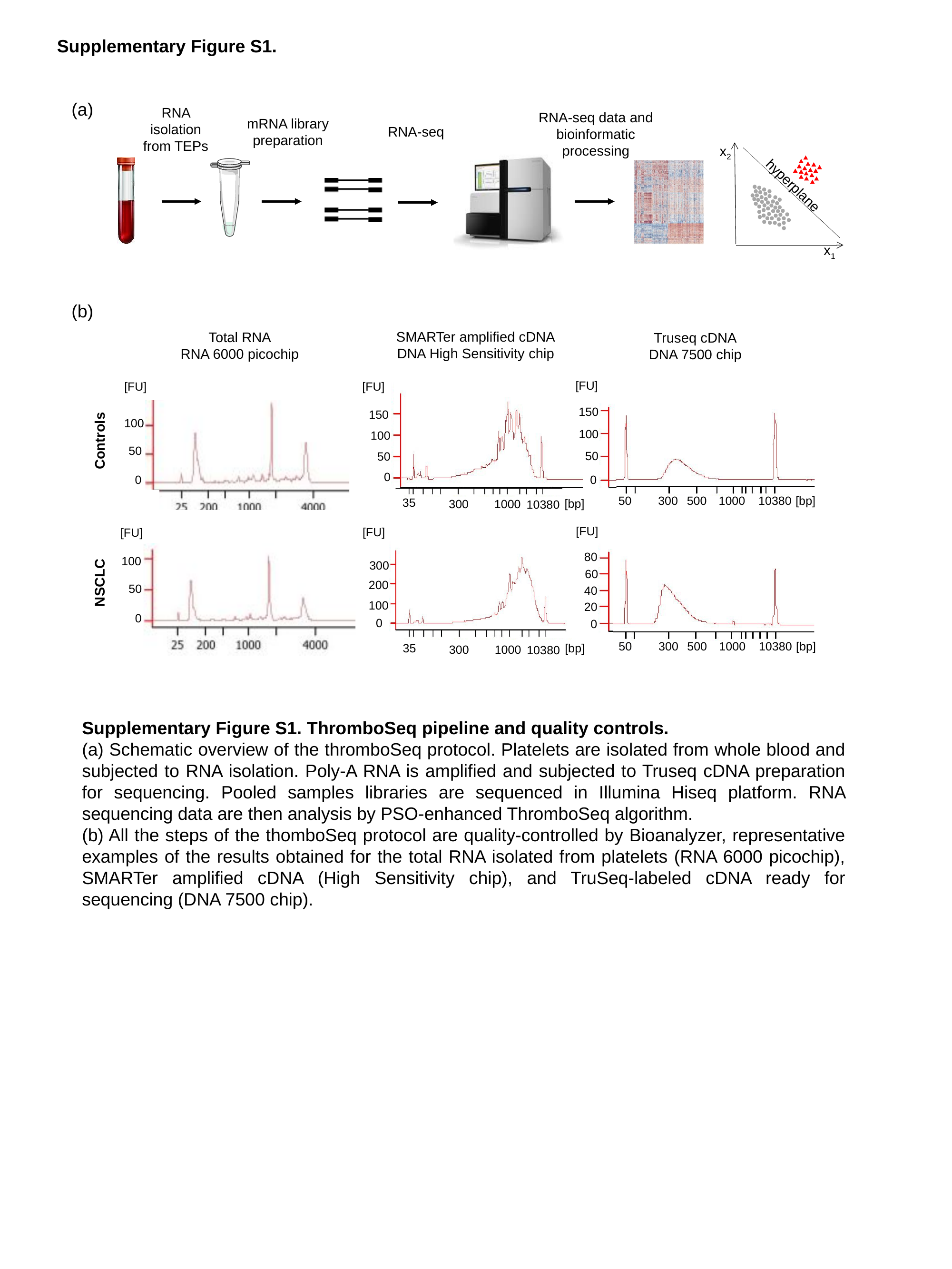

Supplementary Figure S1.
(a)
RNA isolation from TEPs
mRNA library preparation
RNA-seq
RNA-seq data and bioinformatic processing
x2
hyperplane
x1
(b)
SMARTer amplified cDNA
DNA High Sensitivity chip
Total RNA
RNA 6000 picochip
Truseq cDNA
DNA 7500 chip
[FU]
[FU]
150
150
100
100
Controls
50
50
0
0
50
300
500
1000
10380
35
[bp]
300
1000
10380
[FU]
[FU]
80
300
60
NSCLC
200
40
100
20
0
0
50
300
500
1000
10380
[bp]
35
300
1000
10380
[bp]
[bp]
[FU]
100
50
0
[FU]
100
50
0
Supplementary Figure S1. ThromboSeq pipeline and quality controls.
(a) Schematic overview of the thromboSeq protocol. Platelets are isolated from whole blood and subjected to RNA isolation. Poly-A RNA is amplified and subjected to Truseq cDNA preparation for sequencing. Pooled samples libraries are sequenced in Illumina Hiseq platform. RNA sequencing data are then analysis by PSO-enhanced ThromboSeq algorithm.
(b) All the steps of the thomboSeq protocol are quality-controlled by Bioanalyzer, representative examples of the results obtained for the total RNA isolated from platelets (RNA 6000 picochip), SMARTer amplified cDNA (High Sensitivity chip), and TruSeq-labeled cDNA ready for sequencing (DNA 7500 chip).

## Slide 2
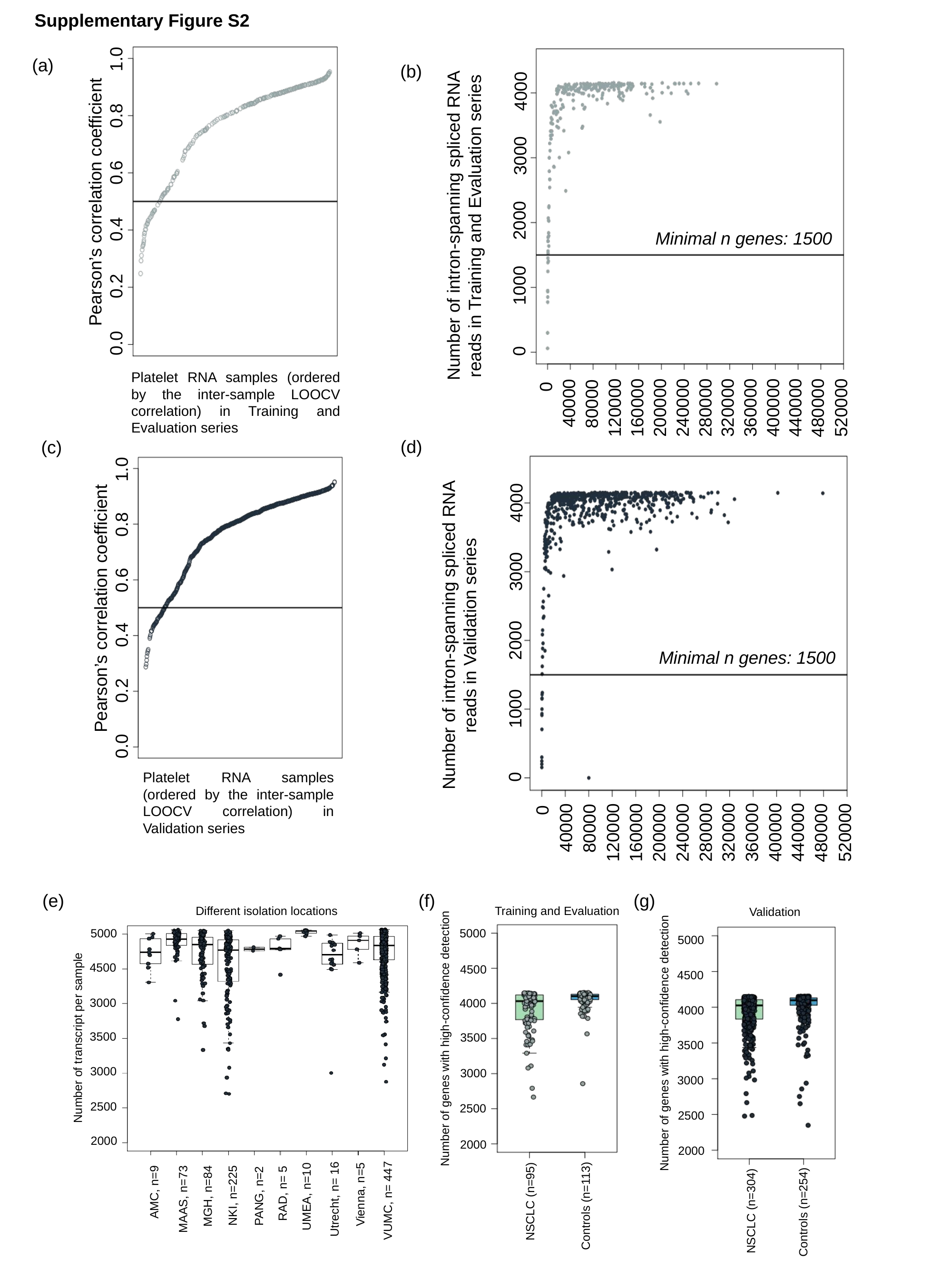

Supplementary Figure S2
1.0
0.8
0.6
Pearson’s correlation coefficient
0.4
0.2
0.0
Platelet RNA samples (ordered by the inter-sample LOOCV correlation) in Training and Evaluation series
4000
3000
Number of intron-spanning spliced RNA reads in Training and Evaluation series
2000
Minimal n genes: 1500
1000
0
0
40000
80000
280000
320000
360000
400000
440000
520000
120000
200000
240000
160000
480000
(a)
(b)
(d)
(c)
1.0
0.8
0.6
Pearson’s correlation coefficient
0.4
0.2
0.0
Platelet RNA samples (ordered by the inter-sample LOOCV correlation) in Validation series
4000
3000
Number of intron-spanning spliced RNA reads in Validation series
2000
Minimal n genes: 1500
1000
0
0
40000
80000
280000
320000
360000
400000
440000
520000
120000
200000
240000
160000
480000
5000
4500
4000
3500
Number of genes with high-confidence detection
3000
2500
2000
NSCLC (n=95)
Controls (n=113)
Training and Evaluation
(e)
(f)
(g)
5000
4500
4000
Number of genes with high-confidence detection
3500
3000
2500
2000
NSCLC (n=304)
Controls (n=254)
Validation
Different isolation locations
5000
4500
3000
3500
3000
2500
2000
AMC, n=9
RAD, n= 5
MGH, n=84
Vienna, n=5
NKI, n=225
PANG, n=2
UMEA, n=10
Utrecht, n= 16
MAAS, n=73
VUMC, n= 447
Number of transcript per sample

## Slide 3
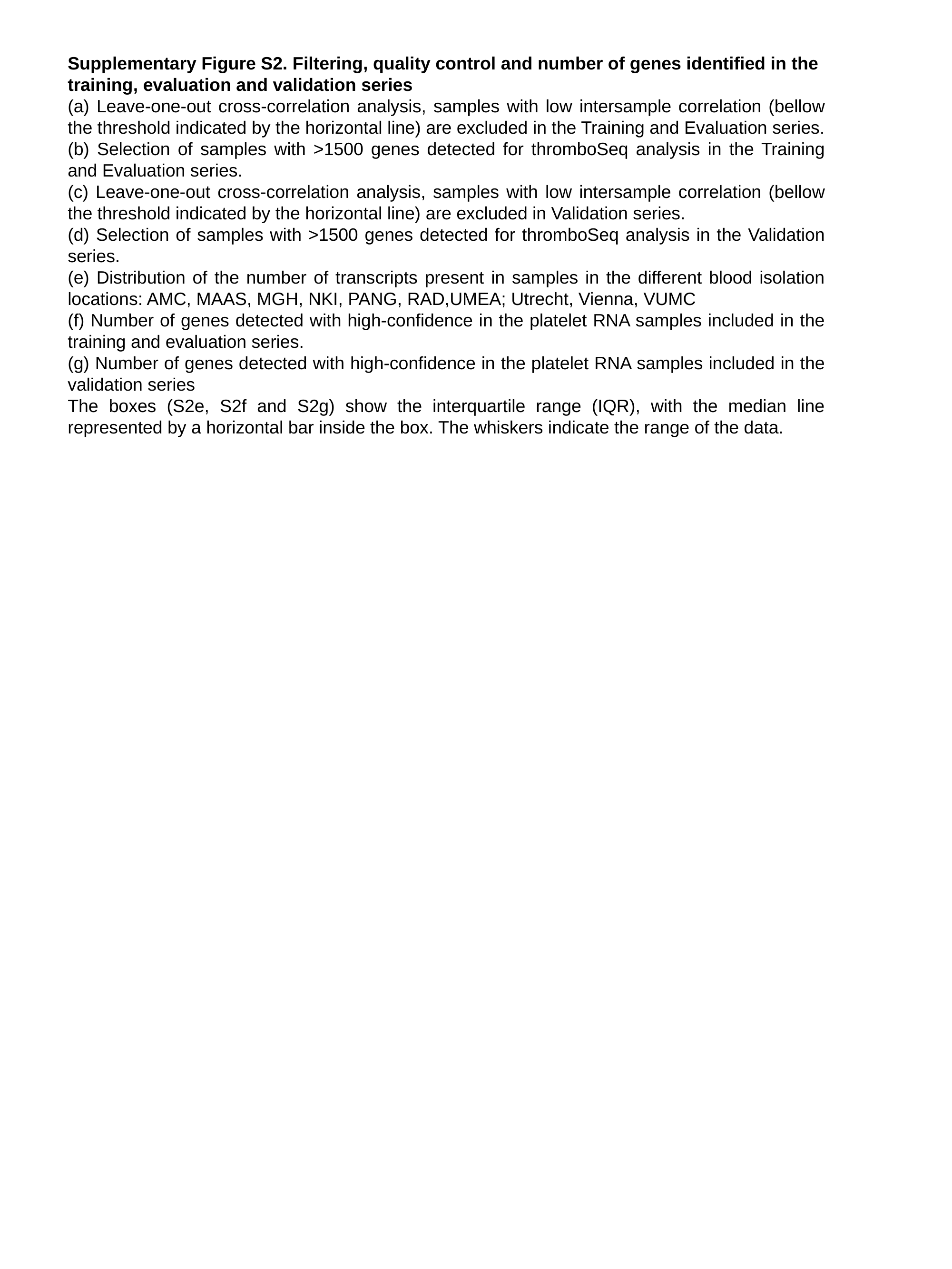

Supplementary Figure S2. Filtering, quality control and number of genes identified in the training, evaluation and validation series
(a) Leave-one-out cross-correlation analysis, samples with low intersample correlation (bellow the threshold indicated by the horizontal line) are excluded in the Training and Evaluation series.
(b) Selection of samples with >1500 genes detected for thromboSeq analysis in the Training and Evaluation series.
(c) Leave-one-out cross-correlation analysis, samples with low intersample correlation (bellow the threshold indicated by the horizontal line) are excluded in Validation series.
(d) Selection of samples with >1500 genes detected for thromboSeq analysis in the Validation series.
(e) Distribution of the number of transcripts present in samples in the different blood isolation locations: AMC, MAAS, MGH, NKI, PANG, RAD,UMEA; Utrecht, Vienna, VUMC
(f) Number of genes detected with high-confidence in the platelet RNA samples included in the training and evaluation series.
(g) Number of genes detected with high-confidence in the platelet RNA samples included in the validation series
The boxes (S2e, S2f and S2g) show the interquartile range (IQR), with the median line represented by a horizontal bar inside the box. The whiskers indicate the range of the data.

## Slide 4
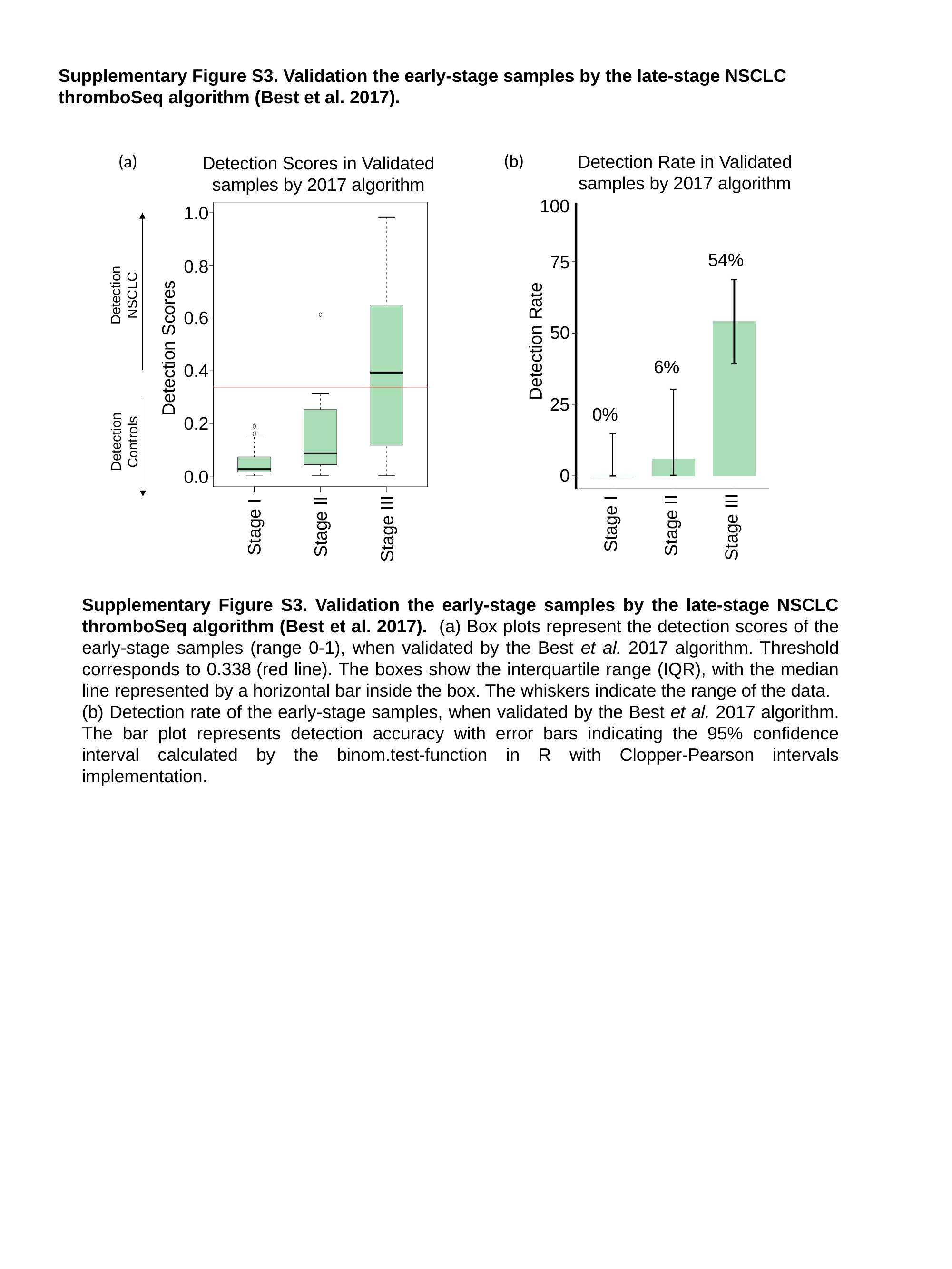

Supplementary Figure S3. Validation the early-stage samples by the late-stage NSCLC thromboSeq algorithm (Best et al. 2017).
(b)
Detection Rate in Validated samples by 2017 algorithm
100
75
50
25
0
Stage I
Stage II
Stage III
54%
6%
0%
Detection Rate
(a)
Detection Scores in Validated samples by 2017 algorithm
1.0
0.8
0.6
Detection Scores
0.4
0.2
0.0
Stage II
Stage I
Stage III
Detection
NSCLC
Detection
Controls
Supplementary Figure S3. Validation the early-stage samples by the late-stage NSCLC thromboSeq algorithm (Best et al. 2017). (a) Box plots represent the detection scores of the early-stage samples (range 0-1), when validated by the Best et al. 2017 algorithm. Threshold corresponds to 0.338 (red line). The boxes show the interquartile range (IQR), with the median line represented by a horizontal bar inside the box. The whiskers indicate the range of the data.
(b) Detection rate of the early-stage samples, when validated by the Best et al. 2017 algorithm. The bar plot represents detection accuracy with error bars indicating the 95% confidence interval calculated by the binom.test-function in R with Clopper-Pearson intervals implementation.

## Slide 5
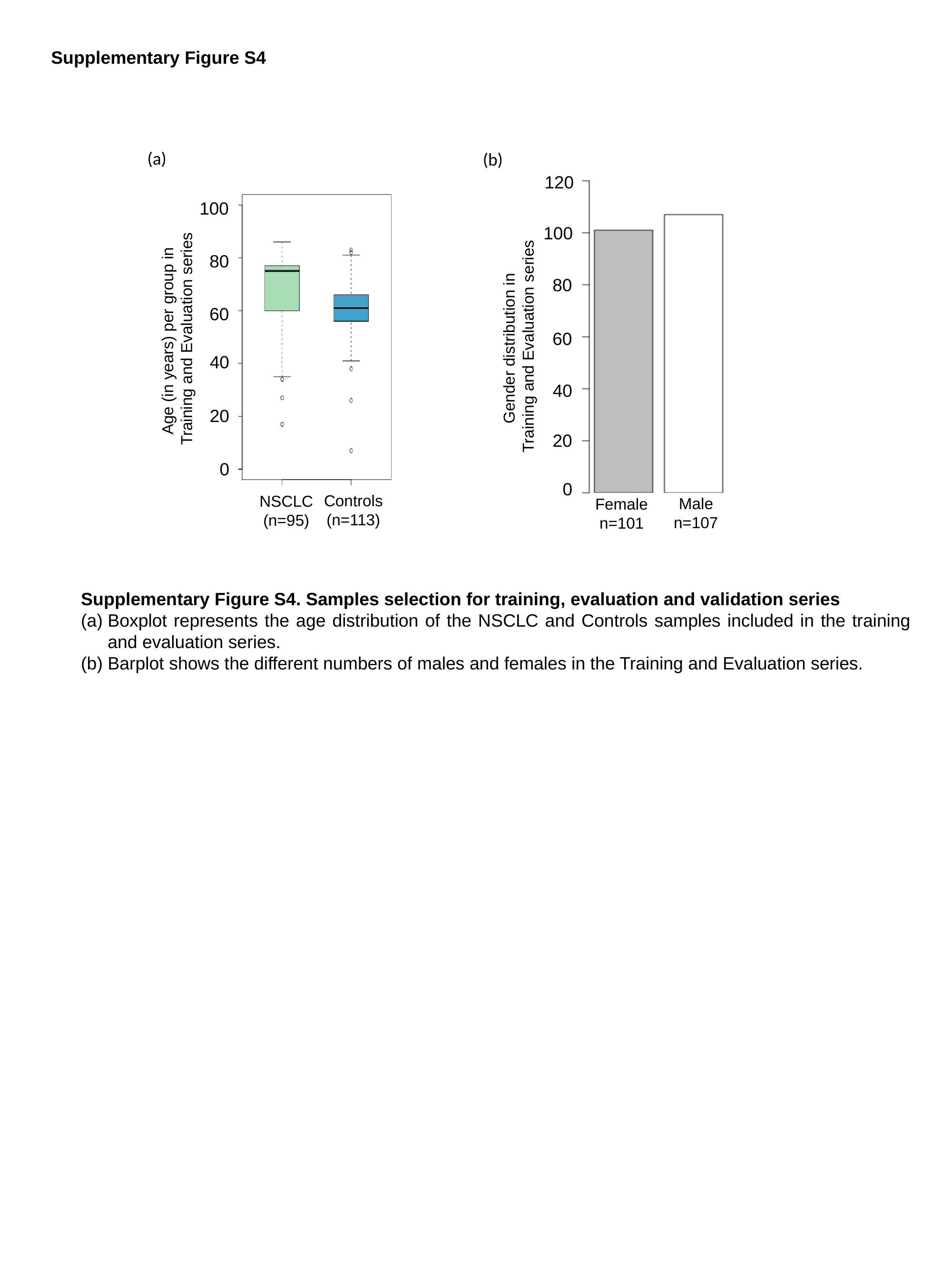

Supplementary Figure S4
(a)
(b)
120
100
80
60
40
20
0
Male
n=107
Female
n=101
Gender distribution in
Training and Evaluation series
100
80
60
Age (in years) per group in
 Training and Evaluation series
40
20
0
Controls
(n=113)
NSCLC
(n=95)
Supplementary Figure S4. Samples selection for training, evaluation and validation series
Boxplot represents the age distribution of the NSCLC and Controls samples included in the training and evaluation series.
(b) Barplot shows the different numbers of males and females in the Training and Evaluation series.

## Slide 6
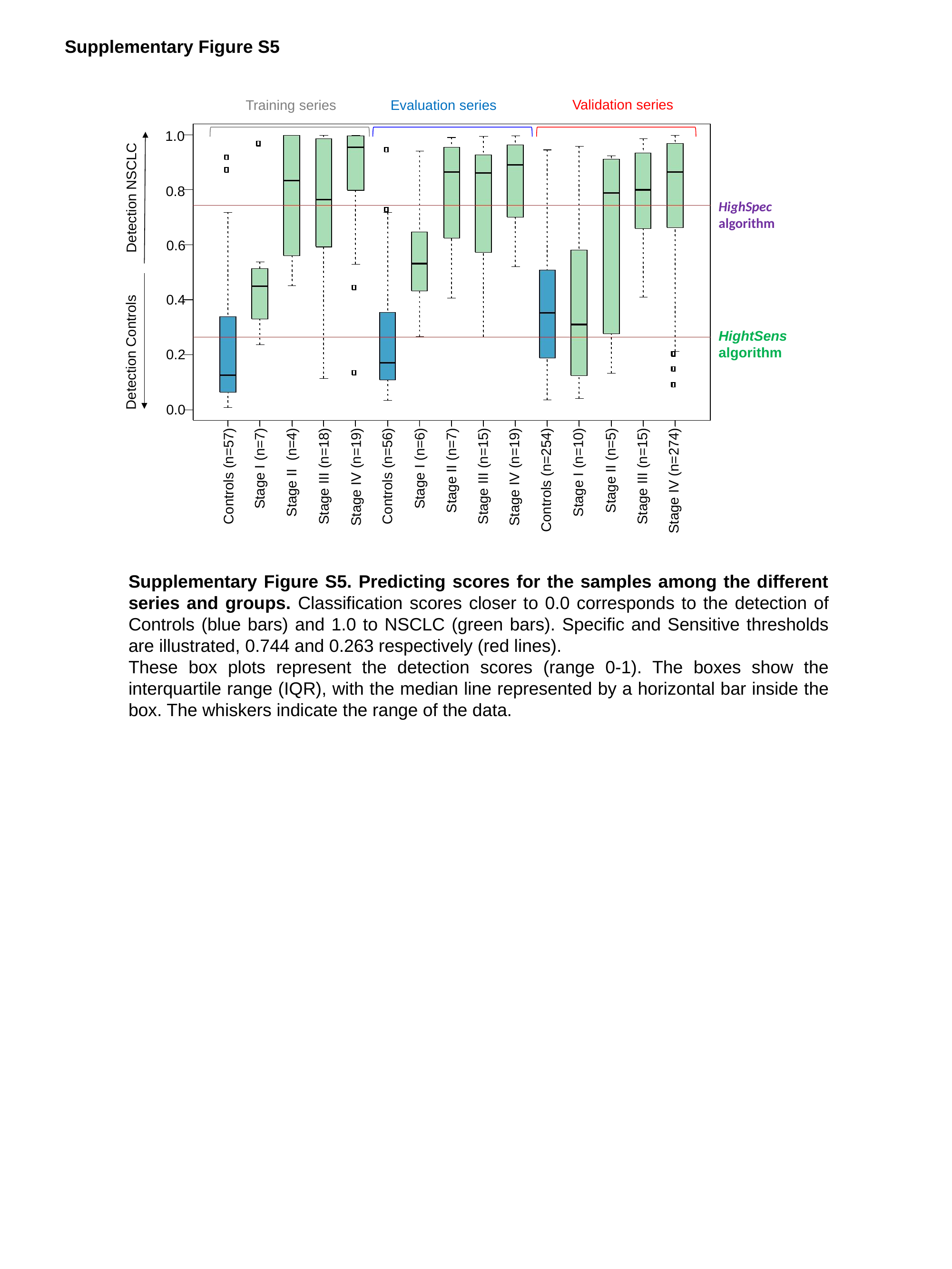

Supplementary Figure S5
Validation series
Evaluation series
Training series
1.0
Detection NSCLC
0.8
HighSpec algorithm
0.6
0.4
HightSens algorithm
Detection Controls
0.2
0.0
Stage I (n=6)
Stage I (n=10)
Stage I (n=7)
Controls (n=57)
Controls (n=56)
Controls (n=254)
Stage III (n=18)
Stage III (n=15)
Stage III (n=15)
Stage IV (n=19)
Stage IV (n=19)
Stage IV (n=274)
Stage II (n=5)
Stage II (n=7)
Stage II (n=4)
Supplementary Figure S5. Predicting scores for the samples among the different series and groups. Classification scores closer to 0.0 corresponds to the detection of Controls (blue bars) and 1.0 to NSCLC (green bars). Specific and Sensitive thresholds are illustrated, 0.744 and 0.263 respectively (red lines).
These box plots represent the detection scores (range 0-1). The boxes show the interquartile range (IQR), with the median line represented by a horizontal bar inside the box. The whiskers indicate the range of the data.

## Slide 7
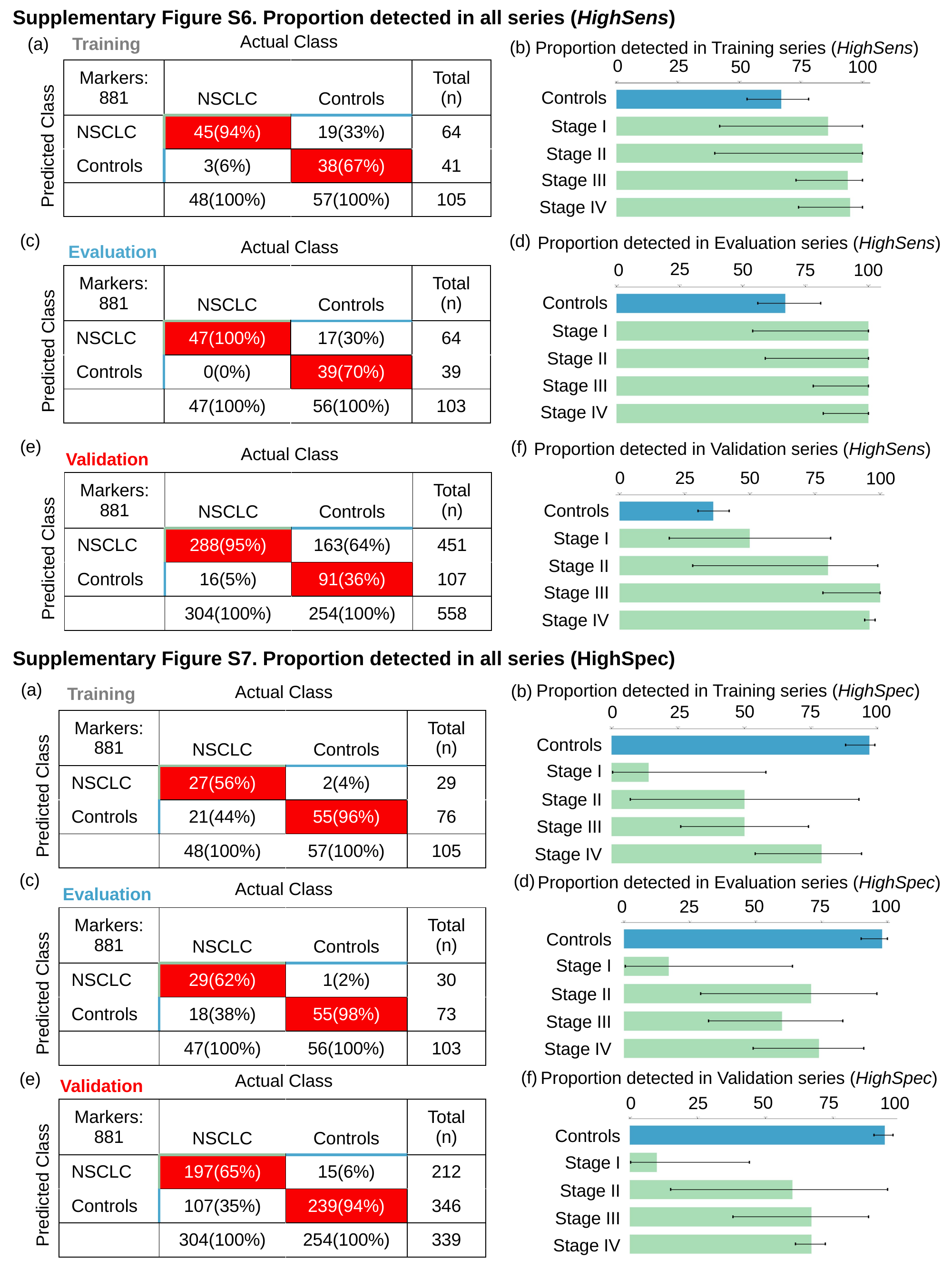

Supplementary Figure S6. Proportion detected in all series (HighSens)
Actual Class
(a)
Training
(b)
Proportion detected in Training series (HighSens)
75
0
25
50
100
Controls
Stage I
Stage II
Stage III
Stage IV
| Markers: 881 | NSCLC | Controls | Total (n) |
| --- | --- | --- | --- |
| NSCLC | 45(94%) | 19(33%) | 64 |
| Controls | 3(6%) | 38(67%) | 41 |
| | 48(100%) | 57(100%) | 105 |
Predicted Class
(c)
(d)
Proportion detected in Evaluation series (HighSens)
25
50
100
75
0
Controls
Stage I
Stage II
Stage III
Stage IV
Actual Class
Evaluation
| Markers: 881 | NSCLC | Controls | Total (n) |
| --- | --- | --- | --- |
| NSCLC | 47(100%) | 17(30%) | 64 |
| Controls | 0(0%) | 39(70%) | 39 |
| | 47(100%) | 56(100%) | 103 |
Predicted Class
(e)
(f)
Proportion detected in Validation series (HighSens)
Actual Class
Validation
25
75
0
50
100
Controls
Stage I
Stage II
Stage III
Stage IV
| Markers: 881 | NSCLC | Controls | Total (n) |
| --- | --- | --- | --- |
| NSCLC | 288(95%) | 163(64%) | 451 |
| Controls | 16(5%) | 91(36%) | 107 |
| | 304(100%) | 254(100%) | 558 |
Predicted Class
Supplementary Figure S7. Proportion detected in all series (HighSpec)
(a)
Proportion detected in Training series (HighSpec)
75
50
100
25
0
Controls
Stage I
Stage II
Stage III
Stage IV
(b)
Actual Class
Training
| Markers: 881 | NSCLC | Controls | Total (n) |
| --- | --- | --- | --- |
| NSCLC | 27(56%) | 2(4%) | 29 |
| Controls | 21(44%) | 55(96%) | 76 |
| | 48(100%) | 57(100%) | 105 |
Predicted Class
(c)
(d)
Proportion detected in Evaluation series (HighSpec)
Actual Class
Evaluation
75
50
100
25
0
Controls
Stage I
Stage II
Stage III
Stage IV
| Markers: 881 | NSCLC | Controls | Total (n) |
| --- | --- | --- | --- |
| NSCLC | 29(62%) | 1(2%) | 30 |
| Controls | 18(38%) | 55(98%) | 73 |
| | 47(100%) | 56(100%) | 103 |
Predicted Class
(f)
Proportion detected in Validation series (HighSpec)
(e)
Actual Class
Validation
75
50
100
25
0
Controls
Stage I
Stage II
Stage III
Stage IV
| Markers: 881 | NSCLC | Controls | Total (n) |
| --- | --- | --- | --- |
| NSCLC | 197(65%) | 15(6%) | 212 |
| Controls | 107(35%) | 239(94%) | 346 |
| | 304(100%) | 254(100%) | 339 |
Predicted Class

## Slide 8
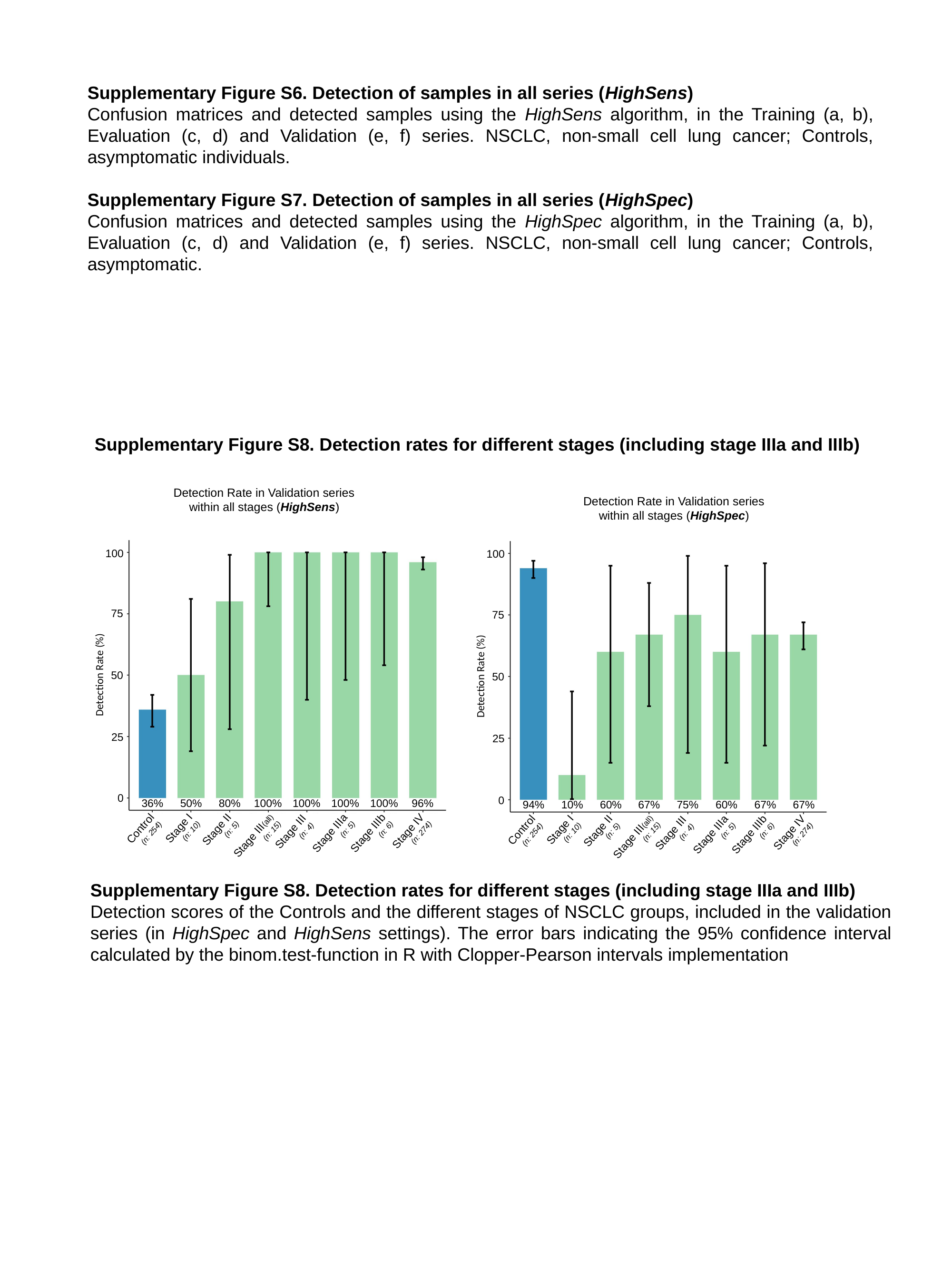

Supplementary Figure S6. Detection of samples in all series (HighSens)
Confusion matrices and detected samples using the HighSens algorithm, in the Training (a, b), Evaluation (c, d) and Validation (e, f) series. NSCLC, non-small cell lung cancer; Controls, asymptomatic individuals.
Supplementary Figure S7. Detection of samples in all series (HighSpec)
Confusion matrices and detected samples using the HighSpec algorithm, in the Training (a, b), Evaluation (c, d) and Validation (e, f) series. NSCLC, non-small cell lung cancer; Controls, asymptomatic.
Supplementary Figure S8. Detection rates for different stages (including stage IIIa and IIIb)
Detection Rate in Validation series within all stages (HighSens)
100
75
Detection Rate (%)
50
25
0
36%
50%
80%
100%
100%
100%
100%
96%
Stage I
(n: 10)
Control
(n: 254)
Stage II
(n: 5)
Stage IV
(n: 274)
Stage III
(n: 4)
Stage IIIa
(n: 5)
Stage IIIb
(n: 6)
Stage III(all)
(n: 15)
Detection Rate in Validation series within all stages (HighSpec)
100
75
Detection Rate (%)
50
25
0
94%
10%
60%
67%
75%
60%
67%
67%
Stage I
(n: 10)
Control
(n: 254)
Stage II
(n: 5)
Stage IV
(n: 274)
Stage III
(n: 4)
Stage IIIa
(n: 5)
Stage IIIb
(n: 6)
Stage III(all)
(n: 15)
Supplementary Figure S8. Detection rates for different stages (including stage IIIa and IIIb)
Detection scores of the Controls and the different stages of NSCLC groups, included in the validation series (in HighSpec and HighSens settings). The error bars indicating the 95% confidence interval calculated by the binom.test-function in R with Clopper-Pearson intervals implementation

## Slide 9
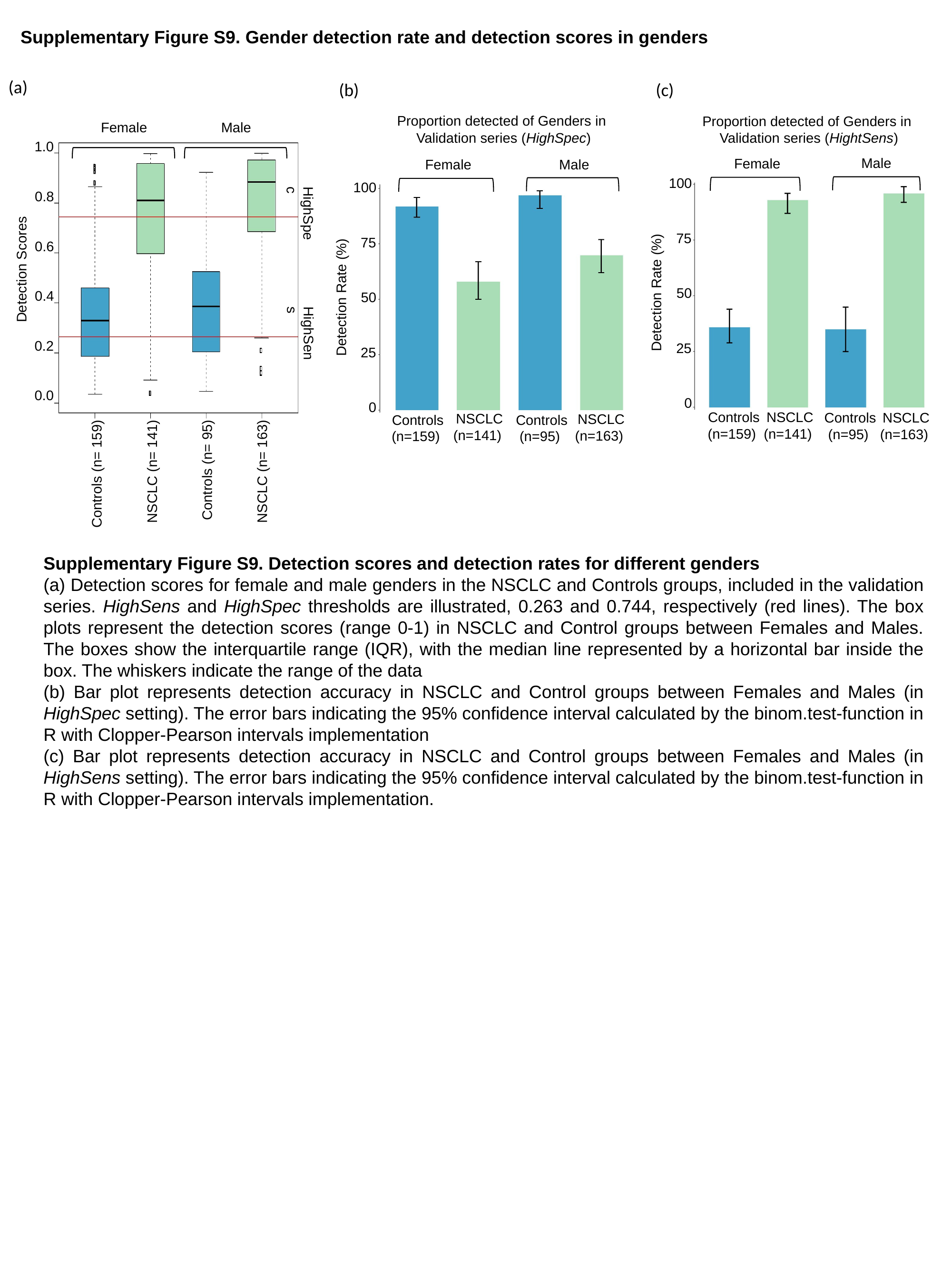

Supplementary Figure S9. Gender detection rate and detection scores in genders
(a)
(b)
(c)
Proportion detected of Genders in
Validation series (HighSpec)
Male
Female
100
75
50
Detection Rate (%)
25
0
NSCLC
(n=141)
NSCLC
(n=163)
Controls
(n=159)
Controls
(n=95)
Proportion detected of Genders in
Validation series (HightSens)
Male
Female
100
75
50
Detection Rate (%)
25
0
Controls
(n=159)
NSCLC
(n=141)
Controls
(n=95)
NSCLC
(n=163)
Female
Male
1.0
0.8
HighSpec
0.6
0.4
HighSens
0.2
0.0
Controls (n= 95)
NSCLC (n= 141)
NSCLC (n= 163)
Controls (n= 159)
Detection Scores
Supplementary Figure S9. Detection scores and detection rates for different genders
(a) Detection scores for female and male genders in the NSCLC and Controls groups, included in the validation series. HighSens and HighSpec thresholds are illustrated, 0.263 and 0.744, respectively (red lines). The box plots represent the detection scores (range 0-1) in NSCLC and Control groups between Females and Males. The boxes show the interquartile range (IQR), with the median line represented by a horizontal bar inside the box. The whiskers indicate the range of the data
(b) Bar plot represents detection accuracy in NSCLC and Control groups between Females and Males (in HighSpec setting). The error bars indicating the 95% confidence interval calculated by the binom.test-function in R with Clopper-Pearson intervals implementation
(c) Bar plot represents detection accuracy in NSCLC and Control groups between Females and Males (in HighSens setting). The error bars indicating the 95% confidence interval calculated by the binom.test-function in R with Clopper-Pearson intervals implementation.

## Slide 10
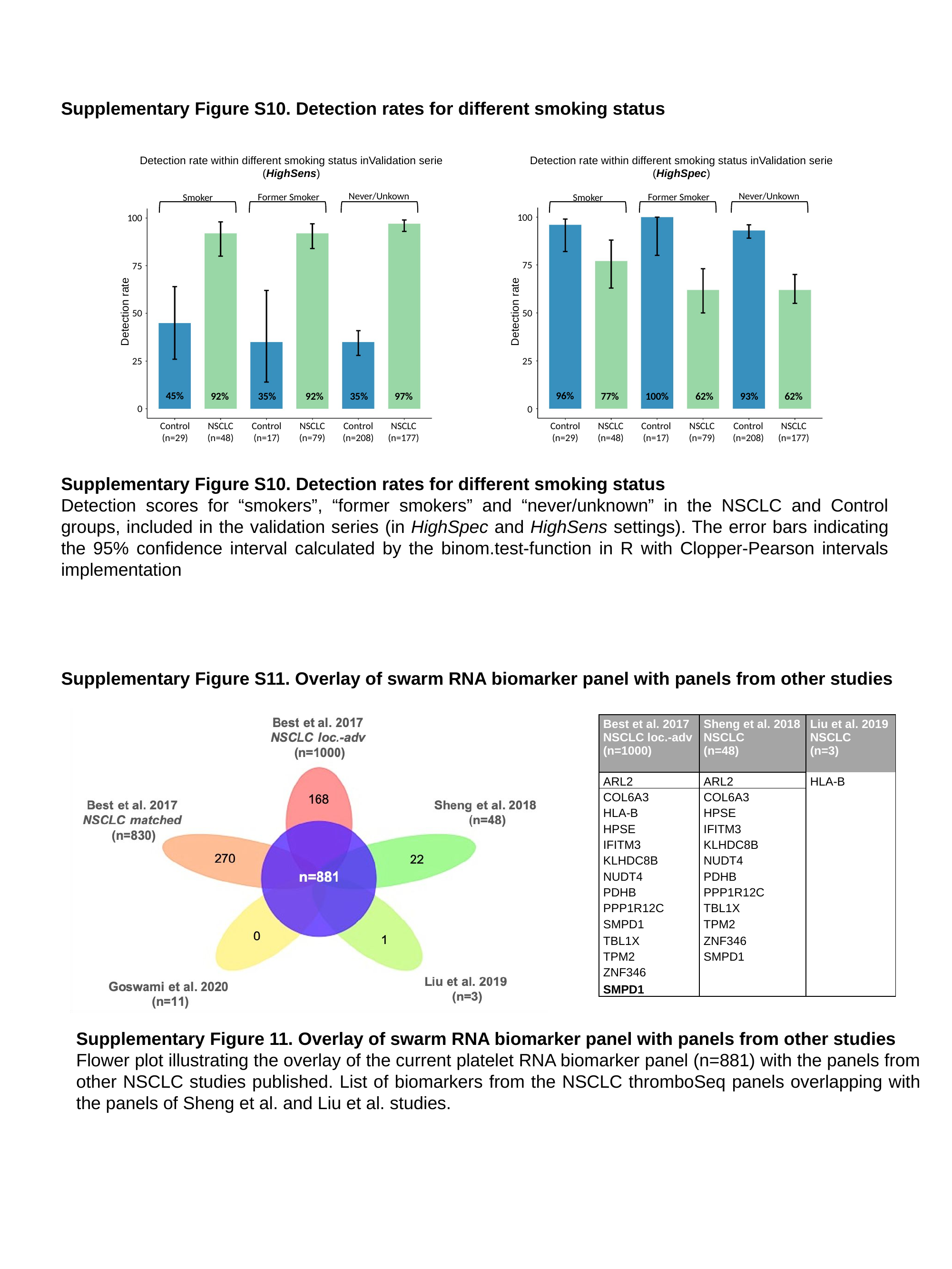

Supplementary Figure S10. Detection rates for different smoking status
Detection rate within different smoking status inValidation serie
(HighSens)
Never/Unkown
Former Smoker
Smoker
100
75
Detection rate
50
25
45%
92%
35%
92%
35%
97%
0
Control
(n=29)
NSCLC
(n=48)
Control
(n=17)
NSCLC
(n=79)
Control
(n=208)
NSCLC
(n=177)
Detection rate within different smoking status inValidation serie
(HighSpec)
Never/Unkown
Former Smoker
Smoker
100
75
Detection rate
50
25
96%
77%
100%
62%
93%
62%
0
Control
(n=29)
NSCLC
(n=48)
Control
(n=17)
NSCLC
(n=79)
Control
(n=208)
NSCLC
(n=177)
Supplementary Figure S10. Detection rates for different smoking status
Detection scores for “smokers”, “former smokers” and “never/unknown” in the NSCLC and Control groups, included in the validation series (in HighSpec and HighSens settings). The error bars indicating the 95% confidence interval calculated by the binom.test-function in R with Clopper-Pearson intervals implementation
Supplementary Figure S11. Overlay of swarm RNA biomarker panel with panels from other studies
| Best et al. 2017 NSCLC loc.-adv (n=1000) | Sheng et al. 2018 NSCLC (n=48) | Liu et al. 2019 NSCLC (n=3) |
| --- | --- | --- |
| ARL2 | ARL2 | HLA-B |
| COL6A3 | COL6A3 | |
| HLA-B | HPSE | |
| HPSE | IFITM3 | |
| IFITM3 | KLHDC8B | |
| KLHDC8B | NUDT4 | |
| NUDT4 | PDHB | |
| PDHB | PPP1R12C | |
| PPP1R12C | TBL1X | |
| SMPD1 | TPM2 | |
| TBL1X | ZNF346 | |
| TPM2 | SMPD1 | |
| ZNF346 | | |
| SMPD1 | | |
Supplementary Figure 11. Overlay of swarm RNA biomarker panel with panels from other studies
Flower plot illustrating the overlay of the current platelet RNA biomarker panel (n=881) with the panels from other NSCLC studies published. List of biomarkers from the NSCLC thromboSeq panels overlapping with the panels of Sheng et al. and Liu et al. studies.
