# Supplementary material for: Tumor-educated platelet blood tests for Non-Small Cell Lung Cancer detection and management
Source: Sci Rep. 2023 Jun 8;13:9359. doi: 10.1038/s41598-023-35818-w (PMC10250384; doi:10.1038/s41598-023-35818-w)
Supplement: Supplementary file 3 — Supplementary Legends. [file 41598_2023_35818_MOESM3_ESM.docx]

**Supplementary Table S1. NSCLC patients and asymptomatic individuals (Controls) characteristics**

For all patients included in this study, the group, tumor stage, gender, age, pathological stage, morphological diagnosis, and location of blood collection is stated. Also, per sample the assigned series for the thromboSeq algorithm training, evaluation or validation is included. Smoking status: current smoker (1), former smoker (2), never/unknown (0). Gender: Male (M), Female (F).
